# Defining Meditation: Foundations for an Activity-Based Phenomenological Classification System

**DOI:** 10.3389/fpsyg.2021.795077

**Published:** 2022-01-28

**Authors:** Terje Sparby, Matthew D. Sacchet

**Affiliations:** ^1^Rudolf Steiner University College, Oslo, Norway; ^2^Department of Psychology and Psychotherapy, Witten/Herdecke University, Witten, Germany; ^3^Integrated Curriculum for Anthroposophic Psychology, Witten/Herdecke University, Witten, Germany; ^4^Meditation Research Group, Center for Depression, Anxiety, and Stress Research, McLean Hospital, Harvard Medical School, Belmont, MA, United States

**Keywords:** meditation, classification, phenomenology, activity, first-person, consciousness, mental activity, taxonomy

## Abstract

Classifying different meditation techniques is essential for the progress of meditation research, as this will enable discerning which effects are associated with which techniques, in addition to supporting the development of increasingly effective and efficient meditation-based training programs and clinical interventions. However, both the task of defining meditation itself, as well as defining specific techniques, faces many fundamental challenges. Here we describe problems involved in this endeavor and suggest an integrated model for defining meditation. For classifying different meditation techniques, we draw on classical, contemporary, and holistic systems of classification. We analyze different techniques and propose that all meditation techniques are based on a specific set of activities, that is: focusing, releasing, imagining, and moving in relation to an object of meditation, including fields of experience. Meditative activities can be combined and unified in the activities of observing, producing, and being aware. All meditative activities are unified in awareness of awareness. Defining specific meditation techniques may be done by specifying which activities and objects are involved. The advantage of our approach is that it can potentially account for the inner workings of all current systems of classification and hence it lays the foundation for formulating an overarching system of meditation that can guide future research and practice.

## Introduction

Classification is fundamental to science (Hoyningen-Huene, 2016). For instance, chemistry is concerned with the classification of different chemical compounds. This is done to get a clear view of what the chemical substances consist of and how they may interact with other chemical substances and other objects or phenomena. In psychology, the most well-known classification system is the *Diagnostic and Statistical Manual of Mental Disorders* (DSM–5) (American Psychiatric Association, 2013). Once a patient has been diagnosed with a mental illness according to the DSM, a clinician may select a specific treatment. Without overarching systems of classification in science there would be no agreement on what basic terms mean, and thus coordinating research efforts toward advancing knowledge would be very difficult or even impossible.

The issue of classifying meditation techniques becomes more and more pressing given the considerable and growing interest in meditation research. To date, it has been shown that different meditation techniques, such as focused attention (FA) and open monitoring (OM) techniques, may lead to both different and overlapping effects (Sears and Kraus, 2009; Perlman et al., 2010; Britton et al., 2018; Zhang et al., 2019). Different meditation techniques have also been shown to lead to different structural changes in the brain (Valk et al., 2017). The fact that there are overlapping effects across different meditation techniques may perhaps be explained by different techniques achieving the same or similar results but through different mechanisms, or rather that different techniques may share *activities* and hence involve the same mechanisms. In either case, the existence of both overlapping and unique technique effects needs to be accounted for. To pursue this kind of research it will be necessary to clearly define and differentiate different meditation techniques.

Furthermore, meditation research is expanding to include possible negative, challenging, unwanted, or harmful effects (Lindahl et al., 2017; Farias et al., 2020; Hirshberg et al., 2020), and it has been shown that some meditation techniques (e.g., insight meditation) may be associated with higher frequency of unpleasant psychological experiences than others (Schlosser et al., 2019). Again, without identifying both similarities and differentiating between and among techniques, such associations cannot be investigated. Furthermore, equating different meditation techniques that may have different outcomes could easily lead to inconsistent research results. To build this intuition, consider researching the effects of different antibiotics without differentiating between unique classes of antibiotics. The results of such research would most likely be quite inconsistent, both highly promising and disappointing at the same time and thus confusing and unhelpful, given that different strains of antibiotics are very effective for certain diseases while ineffective for others (Grayson et al., 2017). Indeed, Goyal et al. (2014), while calling into question the quality of meditation research, notes that “[u]ncertainty remains about what these distinctions [between mindfulness meditation, concentration, and automatic self-transcendence] mean and the extent to which these distinctions actually influence psychosocial stress outcomes.” (p. 358). Clearly and consistently differentiating between meditation techniques is not only an important step to improve the overall quality of meditation research but also an essential part of its scientific maturation. Furthermore, agreeing on a universal framework of definition and differentiation is an important step toward going beyond the confines of the different meditative *traditions*. Though, as we will argue, the context of meditative practice may be vital to gain a full understanding of its meaning, there are also aspects of it, such as the acts that are performed, which may be understood conceptually, experientially, and physiologically without referring to any specific tradition. Clearly, this is a very important development for the modern application and understanding of meditation training.

Two concepts are central to our approach: activity and phenomenology. Regarding the concept of activity, we draw on recent work on mental action in philosophy in relation to meditation. Strawson (2003) has defined mental action as bringing content to consciousness (as in imagining, thinking, and choosing), there are evidently other mental actions as well, such as clearing the mind of content during meditation (Upton and Brent, 2019). Furthermore, we prefer “activity” to “action” since “action” implies strong agency. As we will argue, meditation as an activity may involve agency, but also forms of activity in which agency is reduced, such as “effortless doing.” Furthermore, we connect our concept of activity to Aristotle’s idea of *ergon*, that is, an “activity of the soul on the basis of virtue” (Aristotle, 1984, p. I 7, 1098a16–18) or an activity aimed at realizing the human good or the best human achievement (Baker, 2021). In our exposition of the meditation as an activity we view an activity in relation to a beneficial aim and the context of that aim, drawing inspiration from previous work showing that the activities cannot be made sense of fully without viewing it within the context they are enacted (Dahlberg, 2006; Holton, 2009; Noë, 2012). Note also that we do not presuppose that concepts such as “mental” and “physical” can ultimately be clearly distinguished, which is one further reason why we prefer to refer to “activity” rather than “mental action.” As for the concept of phenomenology, we will rely on Lundh’s recent work on this (Lundh, 2020) and pursue a largely Husserlian or theoretical phenomenological approach that investigates the fundamental structures of human subjectivity on the basis on methodological principles such as epoché and eidetic variation (Husserl, 1976; Moustakas, 2010). This approach is based on the notion that research on the effects of meditation should involve investigating what causes those effects. In the case of meditation, the causes are most directly accessible through what meditators *do*, and what they do, considering that meditators are mostly engaged in activities that are not externally observable, will have to be based on phenomenological reports and analysis.

In what follows, we consider several problems with current classification systems (see section “The Shortcomings of Current Classification Systems of Meditation”) and indicate how to improve them (see section “The Way Forward”). We then give an account of differences among classical, contemporary, and holistic classification systems (see section “Classical, Contemporary, and Holistic Systems of Classification”), identify fundamental challenges for an overarching theory of meditation practice (see section “Challenges for Defining Meditation”), and, finally, outline an integrated model of meditation (see section “An Integrated Model”). Finally, we unite the problems and insights of the prior sections into a proposal for a classification of the fundamental meditative activities (see section “Overview of an Activity-Based Phenomenological Classification System”).

### The Shortcomings of Current Classification Systems of Meditation

The need for a classification system of meditation is clear. Despite this, a general consensus for defining and differentiating between meditation techniques currently does not exist. Multiple attempts have been made at classifying meditation. Lutz et al. (2015) have proposed a phenomenological classification of mindfulness meditation. According to this model, meditation techniques can be classified according to the aspects of *object orientation, de-reification*, and *meta-awareness*; the qualitative dimensions of *aperture*, *clarity*, *stability*, and *effort*; and, finally, different *shared contextual features* (Lutz et al., 2015). Schmidt (2014a) has proposed a comprehensive system of classification ordered according to four main categories: *attention regulation*, *motivation*, *attitude*, and *practical context*. Recently, an empirically derived system of classification for meditation has been proposed by Matko and Sedlmeier (2019). Matko and Sedlmeier (2019) initially identified 309 different meditative techniques based on handbooks and research articles. Among these they chose the 20 most popular techniques and asked 100 expert meditators to rate the similarity among these techniques. This became the basis for developing a model for classifying meditation techniques according to *activation* and *amount of body orientation*. This was followed up by a study of what meditators do when they meditate. In this study Matko et al. (2021) attempted to create a comprehensive list of techniques derived from the initial 309. Following a systematic procedure based on, for example, combining similar techniques and removing vague ones, the initial 309 techniques were reduced to 50. The list of 50 techniques was presented to 635 expert meditators asking them whether they had experience with these techniques and whether any techniques should be added. The list was found to be adequately comprehensive, with only two further techniques (“sitting in silence” and “expressive practices” added to the list of 50) (Matko et al., 2021, p. 1797).

These classification systems have several shortcomings. One may ask whether the proposed definitions of meditation have been too wide, for example, if we include the aspects mentioned by Schmidt (2014b). An individual who regulates attention based on a specific motivation and attitude within a practical context may be performing progressive muscle relaxation, or other techniques that may not be strictly meditation because their primary aim is to relax rather than to heighten and cultivate awareness toward “awakening.” In other contexts, the definition of mediation may be too narrow. For example, Lutz et al. (2015) only sought to classify *mindfulness* meditation and hence their model must be extended if it is to count as a universal system for classifying meditation. Furthermore, engagement with the traditional and contemporary meditation handbooks is limited, as evidenced by statements such as “all meditation techniques have a somatic component and meditation is inherently embodied” (Matko and Sedlmeier, 2019, p. 10). Such statements disregard meditation states that *are not necessarily embodied*, such as deep states of meditation, sometimes referred to as jhāna, in which there may only be pure light present, and the formless jhānas (Catherine, 2008; Snyder and Rasmussen, 2009; Yates et al., 2015). The engagement with meditation handbooks is important not only because it offers a better understanding of meditation practice, but also because such books contain descriptions of deep states (Reddy and Roy, 2018, 2019a,b,^2020^). Although such states may be uncommon, they may nonetheless be significant as they represent potential realizations of the more advanced aims of meditation practice. In addition current systems of classification may not adequately differentiate between meditation techniques. For example, none of the previously proposed systems differentiate between meditation that may be practiced with eyes open, eyes closed, or different degrees of partially closed eyes. Previously proposed models also fail to specify fundamental changes of the meditative activity such as when the practice becomes non-intentional. Furthermore, they are limited in accounting for different postures (e.g., walking and sitting meditation, including different sitting postures). Finally, the classification systems are not able to account for differences in specific variations to techniques such as focusing on sensations in specific body regions, and they fail to account for how all possible variations of meditation techniques relate to an underlying principle of what meditation is. To summarize, the existing meditation classification systems: (1) either define meditation too widely or too narrowly; (2) fail to provide a way of classification that may adequately distinguish between techniques; and (3), most importantly, do not explain the connection between different aspects of meditation and the underlying principle of what meditation *is*. Advancing a scientific system of classification of meditation requires that we overcome these limitations.

These criticisms are, however, not intended to say that these previously proposed systems should be abandoned entirely. Rather, after having considered such criticisms, the different classification systems should be reviewed and synthesized, which may lead to a truly comprehensive account of different meditation techniques. Before that can take place, however, we suggest that including *phenomenological methods* is vital to understanding meditation. We argue that a phenomenological approach will provide an account of the relationship between what meditation *fundamentally* is, in addition to the different techniques that are related to it. We believe that the approach of developing classification systems that are empirically based through investigating the activities of meditators, as proposed by Matko et al. (2021), is consistent with our approach, even if preliminary.

If an effect is experienced during meditation, it is most likely associated with preceding or ongoing changes within consciousness, and the most central change that takes place when someone meditates is related to what activities they perform. However, a fundamental problem is that certain forms or techniques of meditation often, if not always, involve more than one activity. For example, Upton and Brent (2019) considered the technique of focusing attention on the sensations of the upper lip that is often encouraged in Buddhist meditation handbooks (the sensations include warmth, cold, pressure, tingling, etc.). They identified six different mental activities: (1) introducing and (2) maintaining focus, (3) background awareness, (4) noticing and (5) shifting attention when thoughts appear, and (6) removing mental content (Upton and Brent, 2019). Further adding complexity is that meditative activities may run in parallel. Consider the following descriptions of three meditation techniques from Matko et al. (2021):

1. Being mindful of the rise and fall of the abdomen while breathing.
2. Focusing on the pauses between inhalation and exhalation, carefully observing what happens.
3. Labeling mental experiences with words that describe these experiences.

Rather than being techniques that necessarily exclude each other, each of these meditations can be performed in parallel, and such combinations are even recommended in influential meditation manuals (Yates et al., 2015; Sayadaw, 2016). Variations among techniques may indeed be essential to ensure progress at specific stages of practice (Yates et al., 2015; Sayadaw, 2016; Reddy and Roy, 2019b).

Two important points can be made from these observations. First, given that the proposed meditation techniques do not exclude each other, how can they be called *different* techniques? According to traditional ways of creating taxonomies two species must be differentiated in a way so that they logically exclude each other (see section “Classical, Contemporary, and Holistic Systems of Classification” below). Second, if the practitioner is meditating according to either of these techniques, attention will eventually drift away from the meditation object or the meditator will inadvertently discontinue the meditation task (for instance as thoughts appear). At that point, the practitioner could either react as quickly and energetically as possible and return to the meditation object of focus, or one could relax inwardly, practice non-judgment, and return to the object slowly. These additional activities are clearly part of the meditation process itself and examples of opposite approaches of engaging with attention (i.e., increasing vigilance forcefully or reducing stress to the system while supporting mindfulness). Such differences in approach are highly likely to influence the effects, *or lack thereof*, that meditation has. Said another way, there needs to be a systematic method to clearly defining meditation techniques in a manner that they are adequately differentiated from one another. All activities involved in a technique need to be specified.

## The Way Forward

In section “The Shortcomings of Current Classification Systems of Meditation” we highlighted the level of analysis that we believe meditation research requires in order to develop an accurate and comprehensive classification of meditation. In our view, meditation research has yet to uncover what characterizes meditation practice as such, especially the specific elements that constitute different meditation techniques. Several studies have already shown that there are several micro-gestures, that is, quick, prereflexive mental acts, involved in meditation, and the acts people perform during meditation, for example, when they begin and cease to mind-wander, may be different (Petitmengin et al., 2017; Przyrembel and Singer, 2018; Sparby, 2019c). To ensure the validity of meditation research, it is vital not only that the names of meditation techniques are agree upon, but also that the *actual activities* performed are identified. Otherwise, investigators risk either conflating techniques or failing to recognize that they are related: One may think that two individuals are performing the same technique, while, on the level or activity, they are doing something quite different. Or two individuals who label their techniques differently may rather be performing the same, or similar, activities. Furthermore, the mechanisms underlying why some practitioners experience beneficial effects from meditation, while others do not, could be related to performing, or failing to perform, several different activates and micro-gestures that are vital for specific beneficial effects to occur.

One approach to creating an initial overview of the potential types of meditations is to analyze meditation instructions. These can come from both traditional sources (Kunga Tenzin, 2014) and contemporary descriptions of meditation practice, such as normative descriptions given by teachers or expert practitioners, guided meditations, and practice reports. We have attempted such a basic classification of the description of the 50 different kinds of meditation that were identified and described in the comprehensive list created by Matko et al. (2021). A basic challenge is to specify exactly which activities can be meaningfully combined to constitute a single category. Furthermore, as already noted in section “The Shortcomings of Current Classification Systems of Meditation,” several different activities can be identified and each of these activities can be combined not only with several different meditation objects or domains of experience, but the activities themselves may also be combined. The number of meditation objects can, in principle, be extended infinitely. While the actual meditation object may be considered trivial or less important, traditional literature does indeed consider different meditation objects to have different effects (Bryant, 2009; Buddhaghosa, 2010). For example, discerning the properties of different body parts may lead to heightened concentration, but it cannot lead to deep states of absorption such as jhāna, which rather requires other objects such as the breath (Catherine, 2011, p. 197).

However, it is not only necessary to analyze the different activities involved in meditation. We must also expand the investigation to include the *context* of meditation, that is, the level of the human lifeworld, traditional frameworks, and philosophical principles. First, to fully understand a human activity one needs to understand the context of the given activity. Imagine two individuals fully immersed in allocating attention toward an object. In this scenario both individuals are focusing attention with the intent of achieving a specific goal, i.e., they are not simply immersed in an experience (like when watching a move). It might seem that both are performing meditative activities. However, as it turns out, one individual is aiming a rifle at an animal with the intent of making a kill, while the other is watching their breath with the intent of increasing concentration toward understanding reality more deeply.

Clearly, the context of the activity plays an important role in defining the activity. Although the activity of attending to an object may be similar, what one (ultimately) *intends* to achieve by closely attending to experience cannot be fully separated from the activity itself, as indicated in the introduction in relation to Aristotle and pointed out in related contexts by other researchers (Dahlberg, 2006; Holton, 2009; Noë, 2012). Furthermore, to understand an activity more completely, one needs to understand other aspects of the context as well. For example, in relation to the above scenario, understanding what an animal is, and a rifle or reality and truth. Indeed, meditation can involve the deepest ontological and metaphysical questions human beings ask: What is the nature of being? What are the principles that rule phenomena? What is the self? For example, some meditations involve investigating the essence of the mind, asking whether it is existent or non-existent. Other meditations involve investigating the essence of external phenomena, for example, aiming to realize that the mind and the world are not separate.

Whether or not the ultimate aim, or aims, of meditation are realizable is not a question that needs to be answered for developing a classification system. However, to fully understand an activity, its aims still need to be considered. For example, understanding that pressing a big red button will initiate an atomic war is essential to understanding what pressing that button actually consists of. It could be said that someone is not guilty of starting an atomic war if they did not know what pressing this button leads to, but that does not mean that pressing the button does not mean starting an atomic war. Similarly, someone performing a body scan meditation during a Mindfulness-Based Stress Reduction (MBSR) course may come to realize the non-existence of the self, the impermanence of all phenomena, and/or that suffering is all-pervasive, as such realizations are among the ultimate aims of such techniques (Anālayo, 2018, 2020). Although one may be unaware of the potential of the realizations that may occur when performing such a technique, it does not mean that the given activity (e.g., body scan meditation) does not lead to particular outcomes (e.g., insight into the nature of experience). It might be the case that a given activity requires multiple components. For example, a body scan meditation may involve at least two activities if it is to lead to realizations: observing sensations and becoming aware of how they are impermanent, not the self, and unsatisfactory. Such questions may be regarded as open research questions. The self, impermanence, and suffering are notions that hardly can be considered fully without understanding each of them within the context of the human lifeworld, that is, the world of given everyday experience.

However, although context is necessary for understanding an activity, some separation between the activity and the context is still possible. If meditation required already fully knowing the essence of the mind, external phenomena, etc., then we would *not need* meditation. When we compare two activities like meditative focus and aiming a rifle, there may very well be similarities, for example, on a physiological level. The way forward for a classification system of meditation consists of both understanding the activity *and* context of meditation. Still, as noted, an activity and its context may be treated in some isolation from each other. The performance of an activity has a phenomenological dimension beyond what it aims at. Clarifying the context of meditation involves deep and extensive reflection on topics such as “awakening” or other meditation endpoints. Here we will mostly limit ourselves to classifying the activities involved, well aware that a deep dive into such topics will be necessary to gain a comprehensive understanding of the involved activities.

## Classical, Contemporary, and Holistic Systems of Classification

One can distinguish between classical, contemporary, and holistic systems of classifying meditation. A classical classification system orders *genera* and *species* according to common and differentiating properties or markers. Defining something, according to Aristotle, consists of stating the proximate genera and the species of something (*genus proximum et differentia specifica*). A common marker of a genus must be found in all the species, while the differentiating markers can only be found in the species. A marker that differentiates a species can be said of that species and must be denied of the other species. This means that the species are contradictory with regards to their differentiating markers. Such a method of classification has remained influential and is used in the classification of animals. For example, the definition of the domestic dog by Linnaeus is *canis familiaris*, where *canis* is the genus, and *familiaris* the species. According to Linnaeus, the common marker of *canis* is having 42 teeth (common to both wolfs, *canis lupus*, and dogs). The differentiating marker for the domestic dog is the *cauda recurvata*, the upward turning tail. This means that you cannot predicate the differentiating marker (upturning tail) of a wolf without contradiction. While there may be other features that distinguish between a wolf and a dog, classic systems of classification require that one specific feature is selected. Genera/Species-based classification systems will result in a branching tree structure sometimes referred to as a Porphyrian tree. These kinds of classification systems have the advantage that they are simple and clear. The primary problem with such systems is often that nature is not so simple and clear as the classification system requires. For instance, there is ongoing debate regarding whether a dingo belongs to its own species or rather to *canis familiaris* (Smith, 2015) – and how should one define a wolfdog, a mix between a wolf and a domestic dog? Additionally, common markers may appear to be arbitrary – what is the necessary connection between the inner or essential nature of an animal and the number of its teeth?

For entities that are simpler than living creatures, such as geometrical figures, classification is easier. This is because the nature of the geometrical figure is fully explicated in its definition. However, when it comes to psychological phenomena, such as psychopathologies, the task of classification is considerably more difficult. According to the DSM, psychopathologies are classified according to a set of characteristics (American Psychiatric Association, 2013). If a person exhibits a number of those characteristics, sometimes over a set period of time, they may be diagnosed (classified) as having a certain psychopathology. This *contemporary* approach affords balance between the need to identify psychopathology (which may be important for choosing treatment and coordinating research endeavors) and recognizing that there is some degree of arbitrariness within the classification system (which is updated from time to time based on ongoing research, critique, and dialogue).

Both holistic and non-holistic systems may consist of wholes and parts. However, in *holistic* systems, not only are the parts contained in the whole, the whole is contained in the parts (Bortoft, 2013). Such holistic systems may seem contradictory. If a whole is contained in the parts and the parts are contained in the whole, then the whole is contained in itself. This may seem impossible. If something is to contain itself, it needs to be both separate from, while also part of, itself. A cup can contain water, but a cup cannot contain itself. However, certain things, like self-consciousness and the concept of a concept, are characterized exactly by exhibiting self-containing relationships: in self-consciousness, that which one is aware of is that which is aware. If we take “aware of” to mean “contain” – and why should we not? If I am aware of a cup, the cup is within my consciousness – then it is certainly possible for something to contain itself. Though some will deny that a subject can be made into an object, the experience of self-consciousness or self-awareness shows that it is possible. This is an example of a dialectical relationship between subject and object (someone being conscious of something) ending in a unity of opposites (self-consciousness). Furthermore, as noted above, in classical systems, the *differentia* cannot be contained in the genus, otherwise, one would also get entangled in a contradiction. *Canis*, the genus, cannot have both upward and downward pointing tails but need to be indeterminate in relation to this. This means that genera are more abstract (indeterminate) than their species. However, as Hegel (1834) has shown, basic concepts, such as “becoming,” contain the species “being” and “nothing” within itself in the shape of “coming-to-be” and “ceasing-to-be.” In contrast to classical systems, as the wholes (the genera) of holistic systems contain the differentiated markers in them, they are more concrete (determinate) than their species (Sparby, 2014). As a result, in holistic systems: (1) all parts can be derived from the whole; (2) the whole can be derived from all the parts; and (3) all parts can be derived from all parts. In this way, holistic systems are thoroughly unified.

The classification system for meditative activities outlined below in section “Overview of an Activity-Based Phenomenological Classification System” will draw on all of these approaches to classification: from the classical system the ideal of clarity and simplicity are adopted. We develop from the contemporary approach the recognition that empirical phenomena may to a certain extent be arbitrary, and that a classification system needs input from many different, and continually evolving, scientific disciplines. From holistic systems of classification we integrate the idea of dialectical relationships and that wholes may consist of opposites. For clarity and simplicity, we will suggest a branching tree structure that is emblematic of classical systems. For empirical basis and input from other disciplines, we will suggest a neuroscientifically informed approach and defining specific techniques according to the emphasis of the practice. For the definition of overarching meditation techniques, and to create a unified account of complex meditative activities, we will draw on the principles of holistic systematicity.

## Challenges for Defining Meditation

Before we attempt to classify different meditation techniques, it is necessary to identify what is common to all meditation techniques. The aim of identifying the overarching genus is an aim shared by all the classification systems mentioned above – without such identification, classification is not possible. If we consider several recent publications within the field of meditation research, we can start to articulate the confusion that surrounds the definition of meditation. Many investigators state that they study meditation only involving a specific techniques, such as FA (Dasanayaka et al., 2021; Zhang et al., 2021). In other studies different forms of meditation have been lumped together without any justification of the implicit claim that they have something essential in common (Haider et al., 2021; Sumantry and Stewart, 2021). Though there is general agreement that meditation involves many different techniques, attempts have rarely been made to clearly identify common and differentiating markers. Furthermore, there may be some techniques that are not meditative, though they may be similar to those that are. Examples of such techniques include hypnosis, and some forms of intentional visualization and breathing. Making a distinction between meditative and non-meditative techniques presupposes a clear definition of meditation. However, finding a single, clear and comprehensive definition is difficult. This can be illustrated in relation to this attempt at providing a definition of meditation:

Meditation is defined as a mind and body practice focused on interactions between the brain, mind, body, and behavior, containing four key elements: a quiet location with little distractions, a comfortable posture, a focus of attention, and an open attitude. Meditation is often used for its various health benefits, specifically the alleviation of certain mental states, such as loneliness (Saini et al., 2021).

While it seems correct to say that meditation may be a practice focused on interactions between the brain, mind, body, and behavior, this in itself is overly broad: every skilled practice, even eating a meal, or just about anything that a human being does, would be covered by such a definition. Hence, the “key elements” must be essential to the definition. However, a quiet location is not necessary for meditation. In fact, chaotic and loud places can provide the right challenge for deepening practice (Rinpoche and Graboski, 2012). While it may be more difficult to do, for instance, FA meditation under such circumstances, it is not impossible. Furthermore, practicing in a place with distractions may even be recommendable in some cases, such as when one wants to increase required effort. Similarly, though a comfortable posture may work for some, it is neither essential nor recommendable in all cases. Rather, an uncomfortable position may for example represent an opportunity for the practitioner learning about their patterns of reactivity (Didonna, 2020). Furthermore, focusing attention is only representative of certain forms of meditation. Other forms of meditation (such as non-directive meditation or OM) do not involve focusing the mind as such (at least not on something specific, which is what “focus” implies). Having an open attitude may also be essential for certain forms of meditation, but other forms, such as the four immeasurables (loving-kindness, compassion, empathetic joy, and equanimity), concentrate on cultivating specific attitudes (Wallace and Houshmand, 2010). And while it is true that meditation is currently often employed for health benefits, there is usually no mention of what meditation has been traditionally used for. To our knowledge contemplative traditions generally emphasize the training of meditation toward certain endpoints such as “liberation,” “enlightenment,” or “awakening” (Rose, 2016).

Faced with such challenges, it may be tempting to give up on defining meditation in such a manner that would encompass all techniques. Alternatively, one may revert to the stance that meditation is a set of techniques that only have a family resemblance, that is, that they may share features without there being any essence to meditation (Wittgenstein, 1953). Though this might be a valid approach, reverting to a classification system based on family resemblance may fail to discover and identify the basic commonalities, and there are models that provide options beyond family resemblance. Ospina et al. attempted to define meditation using a Delphi process involving seven meditation research experts (Ospina et al., 2007). That is, the experts went through several rounds of dialogue, revising and rating a list of criteria for meditation. Three essential criteria were identified: (1) a defined technique; (2) logic relaxation; and (3) a self-induced state/mode. However, there are several problems with this definition. As Schmidt (2014a) pointed out, these criteria are fulfilled “if somebody plays the guitar in a relaxed mood” (p. 140). Furthermore, there are meditations that *do* involve logical thinking, such as Descartes’s *Meditations*, Zen Koans, and Tibetan style *vipassana* practices. This issue highlights that the second criterion needs to be removed or modified, which would make Ospina et al.’s (2007) criteria for meditation even broader.

However, Ospina et al. (2007) also include mystical experiences, enlightenment, and a religious, spiritual, or philosophical context as an important though not essential criteria of mediation. Considering that meditation traditionally was conceived within such contexts, and aimed at awakening, it is vital that we find clarity regarding this issue – is the context essential or not? Can meditation still be meditation if it is aiming at relaxation? Kabat-Zinn (2018) has claimed that relaxation is not the aim of meditation – non-relaxed sensations connected to frustration and anxiety can be fruitful objects of meditation and not signs that our meditation has failed. Intuitively, it should be clear that an individual maintaining a meditation posture cannot be relaxing completely. But does this mean that the way meditation is typically conceived within contemporary research and within culture at large, as a practice leading to health benefits, is not meditation? Can a technique be detached from its original aim and arbitrarily connected to other aims?

In contemporary meditation handbooks written by spiritual teachers, we find different definitions, or at least characterizations, of meditation that may avoid the problem of bracketing the traditional aims of meditation. For example, Brahm (2014) describes the essence of meditation as follows: “In all types of mysticism and in many spiritual traditions, meditation is the path to a pure and empowered mind” (p. 1). Or, as Catherine (2008) has stated: “Meditation is designed to solve a specific problem: attachment.” (p. 66). Here we see meditation defined according to its aim(s). For Wallace (2006): “Meditation is a balancing act between attention and relaxation.” (p. 32). Here meditation is defined as an act that balances other mental acts or occurrences. Yates et al. (2015) have defined meditation as “the *art* of fully conscious living” (p. xv).

However, some have denied that meditation is an activity, or at least a *goal*-directed activity. For example, it has been claimed that meditation is “not a technique, but a way of being” (Kabat-Zinn, 2018), or, similarly, that “meditation is not an activity conducted while sitting on the pillow but a way of being, a way of living with complete awareness.” (Brown, 2006, p. xxi). Furthermore, meditation may be understood as “*the* way of letting go” (Brahm, 2014, p. 1). Is it possible to actively let go? How can something be achieved, for instance, a certain meditation state, by letting go? Is meditation about actively “training attention and awareness to bring mental processes under greater voluntary control” (Walsh and Shapiro, 2006, p. 228) or “a natural process of coming to rest, [that] requires you to get out of the way completely […] to the point that the process becomes inaccessible to the doer.” (Brahm, 2014, p. 23)? As we see, some scholars and meditation teachers emphasize that meditation involves activities with specific goals in mind, while others reject this notion and do not emphasize *techniques*.

It is clear that defining meditation is indeed challenging when considering all of the various existing characterizations of meditation and their apparent differences. Given the different, and sometimes contradictory, conceptualizations of meditation it may seem that it is impossible to develop a coherent account of what meditation is. Summarizing the above, we highlight here a set of questions, or challenges, that a theory of meditation must overcome in order to answer what meditation fundamentally is:

1. Is it possible to define meditation, or is meditation rather a family of techniques with no identifiable common marker or set of markers?
2. How do we ensure that the definition of meditation is concise and does not include activities that appears to have little to do with meditation?
3. How do we include in this definition the practices that have been traditionally described as meditation?
4. How can we define meditation such that modern research on meditation-related health and other (cognitive, social, etc.) effects can still be included?
5. Is meditation a goal-oriented activity that results in more control of the mind, or rather is meditation concerned with practices of letting go of control and effort?
6. Is meditation a technique or rather a way of being?

Here we can answer these questions tentatively, and the rest of this article will deepen those answers in the manner indicated:

(1) We suggest that although there are different forms of meditation, they have an identifiable common essence. This essence consists of certain common features related to the activity of meditation (see section “An Integrated Model”) as well as a single underlying activity that unifies the more specific features (see section “Overview of an Activity-Based Phenomenological Classification System”).
(2) We can ensure a concise and not overly inclusive definition of meditation by considering the intentionality or the purpose of the practice and its relation to context. While playing an instrument may be similar to certain meditative activities, when it is not done with the purpose of developing, for example, specific skills or traits that ultimately relate to a process of awakening, it is not meditation. However, playing an instrument can be done meditatively. We can differentiate meditative and non-meditative musical performance by considering the mental activities involved and their purpose. For example, one could listen to the disappearing of the sounds with the intention of coming to know the more subtle aspects of reality, the great silence or “supreme word,” paravāc (Padoux, 1992, p. 78).
(3) To ensure that practices that have traditionally been referred to as meditation are included in our definition, it is important to include the *spiritual* perspective, and to consider many different forms of practice from different traditions. This is not an endeavor that will necessarily have to be finished before a definition can be established. There are many texts that need to be considered, and new texts may be found, new traditions may be established or discovered, and the definition of tradition may also change and be expanded. A sign of a useful and generative definition of meditation is its ability to encompass the various technique described in traditional texts. If the definition is not able to do this, it may have to be adjusted.
(4) Meditation can be defined in a manner that includes research on its health benefits by considering the meditative activities both in relation to, and separate from, their spiritual context. Consider the activity of pilgrimage. Pilgrimage may include the activity of walking. Walking has health benefits. Pilgrimage may include experiencing meaningfulness, which may also benefit mental health (Vos, 2016). This does not mean that gaining health benefits by walking while experiencing meaningfulness captures the significance of pilgrimage. In fact, the related health benefits may be seen as secondary or irrelevant, at least for the person participating in the pilgrimage. However, once pilgrimage is undertaken solely for the sake of health benefits, it may be questioned whether it is in fact an authentic pilgrimage. The experienced meaningfulness may be reduced, and the motivation to partake on pilgrimage may lessen. Still, there may be health benefits connected to the physical activity of walking. Similarly, although the activities involved in meditation may have inherent health benefits, meditation cannot be reduced to these effects. The health effects themselves may have to be considered in light of the context, that is, whether it is spiritual or not. Beyond this, it should be obvious that in the same way that the activity of walking within the context of pilgrimage cannot be fully understood without understanding the spiritual aim and context of pilgrimage, so too meditation cannot be understood without considering it within such a context.^1^ Similarly, effects of meditation may be mediated by one’s motivation. For example, an altruistic motivation may be essential for outcomes related to prosociality (Reddy and Roy, 2019a,^2020^). While differences in physical relaxation responses to breath focus meditation may not be significantly impacted by one’s motivation, adherence to practice, emotional responses, and life-impact may very well be. Indeed, motivation may be an important factor when explaining observed limited effects of meditation on prosociality (Kreplin et al., 2018).
(5) and (6) It should be clear that mediation is in some regard a practice, as it lead to certain basic, measurable training effects, such that attention becoming more stable over time (Sumantry and Stewart, 2021). Furthermore, working with intention in different ways (Yates et al., 2015; Sparby, 2019c), and practicing extreme forms of intention realization [such as exiting deep specific jhānas after three hours of absorption without looking at the time (Snyder and Rasmussen, 2009)], is part of traditional meditation practice. When meditation is practiced with a specific health benefit in mind, it is also an example of practicing as a technique with an intention or goal. Furthermore, meditation is motivated in a variety of different ways (e.g., stress-reducation, self-knowledge, and service; Sparby and Ott, 2018), which suggests that for many practitioners it is goal-directed. However, it is also clear that meditation is not always a practice that requires effort. Strong effort may even be detrimental either to achieving the goals or to the practice itself (Yates et al., 2015). One potential issue with effort is that this intention may turn into striving, which is thought to agitate the mind and may therefore counteract effects of meditation. As practice deepens, the meditative activity becomes effortless (Yates et al., 2015; Sparby, 2019c). This does not mean that some balancing of effort and non-effort is no longer required, but rather that the meditative activity has become easier and to some extent self-maintaining, representing a kind of ‘‘flow’’ state.^2^ As meditative expertise develops, the practitioner may experience what is sometimes referred to as non-meditation (Kunga Tenzin, 2014). This is when practitioners “go beyond” acting in one way or another, and meditation rather becomes a continual way of being. This may be understood as activities that had previously required some effort, or subtle activity, to maintain have become habitual or spontaneous.

**FIGURE 1:**
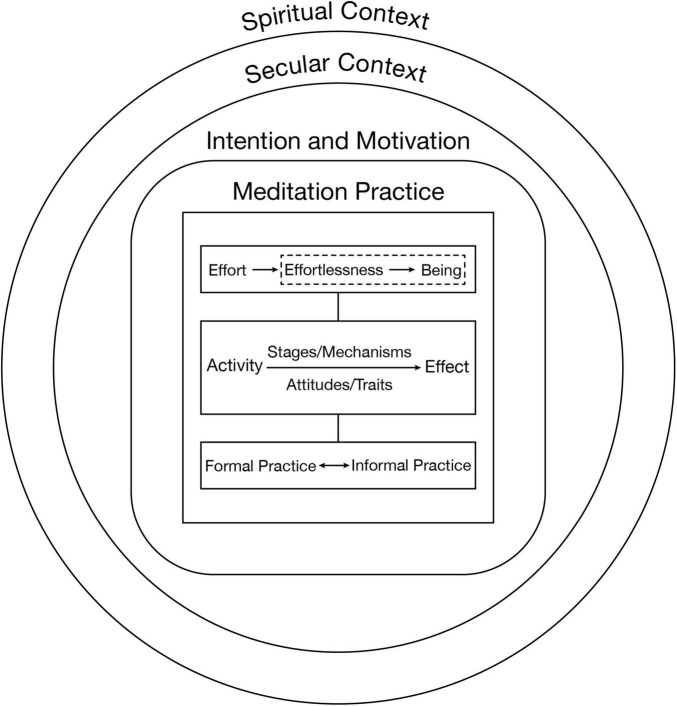
Central aspects of meditation. The figure depicts the main aspects of meditation practice. The rectangular boxes represent aspects of meditation related to the concrete meditative activity while the circles represent the context within which the meditative activity is understood. The rectangular box with curved lines represents the intention and motivation aspect of meditative practice, which connect activity and context. The box with the dotted lines represents a transformation of effort wherein the meditative activity becomes natural and new states of consciousness arise. Further aspects of the figure are explained in the main text.

To define meditation, we believe that it is necessary to combine each of the above elements. Meditation can be defined based on the kind of intentional activities it involves. These activities involve certain mechanisms and effects, and the activities may become effortless and habitual. Although it may be practiced within secular frameworks, ultimately it has to be understood within a spiritual framework. We may represent this definition visually as in Figure 1, which depicts the central aspects of meditation as developed here. This figure is further explained in section “An Integrated Model.” However, as indicated earlier, the essence of meditation is based in the activities performed, which are treated in detail in section “Overview of an Activity-Based Phenomenological Classification System.”

## An Integrated Model

Figure 1 provides an overview of different aspects that are central to meditation as an activity. The primary differentiation depicted in this figure is between the context of the activity and the activity itself. The context concerns everything related to understanding what the meditative activity is *about*, while the activity concerns what is *performed*. The activity can be the same while the context changes. For instance, the activities performed within a meditation session undertaken with the understanding that the practices lead to health benefits do not have to be different from a meditation session done by a more “spiritually” oriented practitioner. We may define spiritual context as that which includes reference to transcendence, the divine, and/or awakening; within a secular context, such aspects of meditation practice are bracketed or denied. In relation to meditation, a spiritual context might include some, but not necessarily all of the following: ideas as awakening (Komarovski, 2015), the dissolution of ego (Lindahl and Britton, 2019), the unification of self with the ultimate being or good (Bryant, 2009; Chlup, 2012), dissolution of fixed concepts and/or the appearance of ineffable concepts or unities of opposites (Sparby, 2015), perceptions of true reality (Sparby, 2019a), the end of suffering (Bodhi, 2000), exalted emotions such as bliss and devotion (Sparby, 2019b), subtle energies (Lindahl, 2017), and the cessation of the cycle of reincarnation or the overcoming of death (Lott et al., 2021). Insofar as a secular context is *not* seen to deny spiritual contexts, they may be seen as complementary. For example, meditation may be understood as including relaxation (see the included Alan Wallace quote cited previously in section “Challenges for Defining Meditation”). Within a secular context, “relaxation” may be understood as a release of muscle tension, a drop in blood pressure, and so on, that is, as something that potentially alleviates negative stress responses and can lead to increased health outcomes. Within a spiritual context, relaxation may be understood as a loosening of habitual, fixed notions that may block the perception of a reality beyond human, cognitive construction. Physiological relaxation responses may be understood in this way as an external appearance of the loosening of a contracted mind and thus complement, rather than contradict, a spiritual perspective.

Between the context and the meditation practice itself is intention or motivation (Sparby and Ott, 2018; Reddy and Roy, 2019a; Sedlmeier and Theumer, 2020). Incorporating intention into the model is vital for distinguishing meditative activities from other non-meditative activities. Meditative focusing, for example, is distinct from unintentional focusing. Meditative focusing includes the intention of remaining focused on the meditation object, and self-correcting when this focus shifts from this object (Latham, 2015). Additionally, intention can be understood in a narrow and broad sense. Intention in a narrow sense refers to what the individual intends to do through the activity (one may intend to focus). In a broader sense, intention concerns the effects that the individual desires. The narrow and broad senses of intention do not exclude each other, but rather the narrow sense is a condition for considering an activity as meditation, at least until the activity becomes effortless. That is, if the intention is not included in the definition, we cannot distinguish meditation from other activities that involve focus on an object, for example, aiming a bow rifle. A meditative activity undertaken without a specific goal is less determinate compared to cases in which the goal is clear. Motivation may be the same as the latter, broad form of intention. Motivation also carries the meaning of having a “motive force,” that is, something that *pulls* or *pushes* the individual toward an aim, for example through inspiration. Any potential effect of meditation, its broader aim, can function as a motivator. It is the meditative activity that is the initial starting point for realizing potential specific effects. Activities and effects acquire full meaning for human life within a specific context. Although the physical activity of walking may the same in different contexts, to human beings it makes a difference whether one is walking to get groceries, going for a leisurely stroll, or is forced to march faster to arrive at a battle in time. The motivation to, or intention for, practice lies between and connects the context and the practice itself. The context may contain a kind of blueprint, a scheme, or an idea of which activities are connected to which effects and why the practice itself is something worthwhile to perform. This blueprint provides a foundation for the development of motivation, which may then drive the practice. The practice is primarily a matter of will, while the context concerns thinking. The motivation contains both will and thinking, as there is an idea that acts as a driver, but also a felt sense of power or conviction (which may vary in strength).

As indicated in Figure 1, the meditation practice itself mainly consists of activity and effect. These effects are mediated by mechanisms and the way an activity unfolds an effect may be categorized into stages. There are two additional important aspects depicted in Figure 1: the notion of effort, and the relationship between formal and informal practice. An activity is usually something that requires at least some degree of effort. Some activities, however, are, or become, effortless. For example, riding a bicycle may be quite difficult for someone just learning to ride. Then, once the individual has gained experience riding a bicycle, it becomes much less effortful. At some point riding a bicycle may even become more or less effortless. Effortless may indeed come in degrees; mounting a bicycle and maneuvering its pedals may require some effort, but once one has gained momentum bicycling is more a matter of relaxing and letting the body do what it already knows how to do, that is, maintaining balance through small, unconscious movements (Sparby, 2019c). For a professional bicyclist, riding a bicycle may become a way of being a matter of one’s being in the world. Everything, including one’s daily schedule, most conversations, and food intake, may be related to the activity of riding a bicycle. In this example, riding a bicycle has undergone a general development from effort, to effortlessness, to being. It is worth noting that effort may again be required during, for instance, competitions, hence, the developmental pattern is “general.” A similar developmental process can be understood in the context of meditation practice. In the early stages of meditative practice – both within a single session and also as one begins a meditation practice for the first time – meditation requires effort. The practitioner must do the activity, become mentally alert, and conduct the task at hand (including releasing any specific activities when doing forms of “non-meditation” or effortless meditation). As one becomes more proficient in meditation, or as the mind habituates to the meditative activities, these practices may become effortless. For example, the meditator’s mind may stay more focused on the object of meditation. When the mind begins to stray, a correction back to the object of meditation may occur spontaneously. As meditative practice deepens, and especially as it starts to seep into daily life, it can become a way of being. The meditator’s thoughts and perceptions, and their interactions, lifestyle, and so on, may all become influenced by, or merge with, the meditative activity.

Meditative activities that have become effortless, habitual, or a way of being may be the key to gaining a better conceptual understanding of deep meditative states. This notion is indicated in Figure 1 by a dotted line around effortlessness and being. With effortlessness and being intentionality changes, that is, in “being” an activity is no longer something that necessarily contains an aim that one intends to realize. Rather, the activity and the aim have become one. For example, in a state of deep concentration (e.g., *samādhi* or *jhāna*) the practitioner does not need to focus because they *are* focus, meaning that the activity of focus is continual and self-correction is no longer needed (Snyder and Rasmussen, 2009). Similarly, in an “awakened state,” fixed conceptions are either continually (habitually, effortlessly, and naturally) dropped or are completely released, though this is more a matter of the informal aspect of practice, which we turn to now.

A typical image of a meditator is of an individual sitting on a pillow. No external movement is observed, while inwardly the individual remains active. After a set period a gong signals the end of the meditation session. This is what formal practice may look like. Formal practice is limited to a time, place, specific forms of embodiment such as sitting, lying, or walking, and one or more forms of meditation. However, the activities performed during a formal session may also be undertaken in daily life. The formal session may prepare, and perhaps condition, the practitioner for performing such activities after the formal meditation has ended. As informal practice is deepened, formal practice is likely to deepen as well. This is because the transition into formal meditation may become less effortful and quicker. Informal practice may also become effortless, or a way of being. As this transition occurs it may give rise to the development of *traits*, or “awakening.” We define a meditative trait as: a habituated disposition to perform meditative activities. Meditative attitudes (e.g., acceptance, being non-judgmental, openness, etc.) may be defined in a similar manner: they consist of a set of activities that are either practiced or have become habituated (at which time they may be regarded as traits).

Secular and spiritual contexts also uniquely interact with both the motivation to meditate, and how effects are understood. For an individual who meditates strictly according to a secular practice perspective, once health benefits or the intended regulation (such as stress reduction or relaxation) is achieved, motivation may diminish. An individual who meditates with a spiritual practice perspective may experience certain effects during meditation, such as the appearance of light or the disappearance of bodily sensations, in meditation (Yates et al., 2015). They may interpret these experiences as indicators to switch the meditation object to either of those experiences in order to deepen practice. Alternatively, an individual mediating based on a secular perspective may understand such phenomena as (perhaps pleasant but also potentially distressing) side effects. Hence, although the meditative activity can be performed independently of an interpretative context, its full meaning arises within such a context, and the meaning itself may strongly impact the practice.

## Overview of an Activity-Based Phenomenological Classification System

The term “phenomenology” is currently used to designate different disciplines. Lundh has – in our view correctly – distinguished between three forms of phenomenology:

(1) theoretical phenomenology, which aims for an understanding of the nature of human subjectivity and tries to delineate basic dimensions of human subjectivity.
(2) Descriptive phenomenology, which uses methods for the description and analysis of people’s actual experiences.
(3) Experimental phenomenology, which aims to study the experiential effects of various phenomenological practices, and to develop phenomenological practice that can have a beneficial influence on people’s experienced life quality (Lundh, 2020, p. 494).

According to this classification, our phenomenological approach described in the subsequent text is theoretical, which has a Husserlian background. However, our approach includes indirect input from experientially based descriptions of meditation (as a way of informing eidetic variation), as indicated below.

Theoretical phenomenology starts with performing the epoché, or the bracketing of one’s natural attitude toward the world. This process includes bracketing all explicit or implicit theories and ideas that one has about the world. In the context of the classification of meditation, one needs to set aside the usual or preconceived conceptions one might have about meditation, including what one practice consists of and aims toward, what meditation is or should be like, etc. Then a phenomenological reduction is performed where the investigator turns to phenomena as they are. In this case this is the activity of meditation. While in principle any meditation may be investigated, to uncover the essence (or possibly essences) of meditation, eidetic variation is necessary. How and how much can we vary the activity of meditation before it stops being meditation? Such a variation may uncover different forms of meditation. However, we need to find a minimal commonality if we are to understand meditation as one essence, rather than a collection of techniques without an inner connection or as a family of techniques with an inner connection that is conceptually inexplicable. Since the imagination of one person can be limited, it can be beneficial to make use of further sources of variation than what one can come up with alone. For the present analysis, we start with the set of 50 techniques described by Matko et al. (2021). We considered all 50 techniques and identified a few basic components, a set of activities and their potential objects. We present these components below. We unified these components within one fundamental essence, that is, the essence of the activity of meditation. This analysis is also not necessarily finished. Regardless, we believe that this system still provides a valuable suggestion and starting point for further investigations, and toward the overall project of comprehensively and definitively classifying meditation techniques.

**FIGURE 2:**
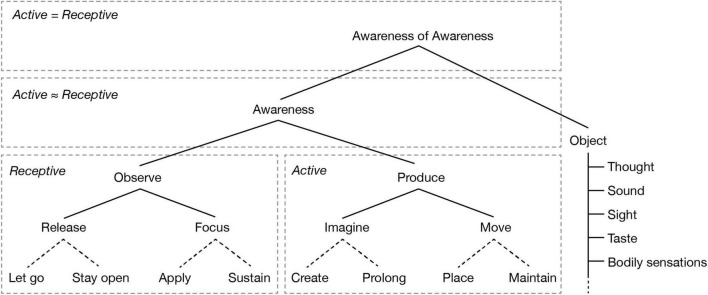
Meditative activities and their objects. This figure depicts a classification system for different meditative activities. Except for the activities connected through dotted lines, the superordinate activities unify, that is share features, of the subordinate activities. The dotted boxes represent different modes of differentiation between active and receptive activities. See the main text for further explanation.

The typical meditation techniques described by Matko et al. (2021), often involve many different activities. In the following our analysis concerns identifying the basic activities that are involved in specific meditation techniques. And later we will group different combinations into classic or well-known techniques. For instructions and examples of how these activities can be used to analyze and define meditation techniques, see Supplementary File 2. Specific meditation techniques can be divided into activities and the objects of those activities, as presented in Figure 2.^3^ Typical objects of meditation include aspects of the breath, the abdomen, other parts of the body, or the whole body, thoughts, feelings, bodily sensations, light, and so on. Meditation objects can also be combinations of one or more of these experiential dimensions, for example, as various impressions of a deity during deity yoga (Eddy, 2019). The list of meditation objects is potentially infinite. Figure 2 only indicates several typical objects, or subordinate domains of objects, that corresponding to several of the senses. This list can clearly be expanded to include additional domains. This is indicated by the dotted line extending from the line connecting the included objects of meditation. Furthermore, each meditation object category may be expanded to include many additional different sub-categories of objects.

Considering typical meditation techniques, we find that some techniques involve *moving* the physical body (walking, dancing, mudras, etc.), maintaining a position, or vocalizing sounds (singing, repeating a mantra). Other meditative activities involve *imagining* something (visualizing, imagining a sound or “energy” flow through the body, etc.). These imagination-based techniques, such as visualization of channels in the body (Worth, 2021), involve using the power of mental representation in relation to the senses or domains of experience of the human being. Other descriptions of meditative practices involve the cultivation of different feelings or attitudes, such as joy and compassion. What each of these meditative activities have in common is that they are all forms of willing or *producing*. These activities all intend to create, change, or in one way or another actively bring about or maintain an object of meditation. These techniques can be understood as varieties of the fundamental activity of *producing*. The subordinate activities are *moving* (for instance the body during walking meditation or when forming a mudra with a hand) and *imagining*. Moving may also be understood to include the activates of placing or continually moving and sustaining a position (like the position of the body). Movement involves imagination in the sense that imagination indicates which movement is to take place. Conversely, imagination implicitly involves movement. That is, movement in the sense of a change from potentiality to actuality, from the “potential to imagine anything” to “imagining something.” In this sense, movement and imagination are unified with the fundamental activity of production.

There are, however, meditation techniques that are fundamentally different from producing, moving, or willing the experience of a mental object. For example, some techniques involve *focus* on an *already existing* object such as the breath, or the observation and mindful awareness of other aspects of our minds. These activities are in a sense passive, or, more precisely, they are *receptive*. If we consider receptivity, we find that it has two aspects. One aspect, *release*, consists of a mind like a *tabula rasa*, so to speak, or letting go of all mental activity. This aspect of release is passive. The other aspect of release, *focus*, is more active. Focus requires an already existing object of meditation. This process is active in that is requires the ongoing engagement of attention. In this way, we can use the active and passive distinction to differentiate between *focusing* and *releasing*. Of note, *focusing* includes activities such as “attending” and “zooming in” (among other activities), while *releasing* can include “relaxing,” “allowing,” “opening,” and “remaining in silence” (among other activities).

*Releasing* and *focusing* can be unified under the superordinate category of *observing*, an activity central to for example the body scan technique (Anālayo, 2020). When the practitioner observes an object, the practitioner is open to it. And, if the object of meditation changes in some manner, the practitioner then is to receive it as it is. The practitioner is to *release* whatever aspect of experience may restrict their access to the object of meditation, such as thoughts and feelings. During observing practices, the meditator also attends to what is observed, though not in a manner focused on a specific aspect of the meditation object (e.g., that the practitioner may “zoom in” on). Hence, with regard to meditative activities, there is a fundamental distinction between *observing* and *producing*. With that said, both are forms of *meditative activities*. And so, we may ask, what do they have in common? And are these two activities not fundamentally opposed, as something active (produce) and passive (observe)? Receptive and spontaneous? We can indeed unify producing and observing. This unification is possible through the concept of *awareness*. When aware of something (without focusing or observing specifically), the individual is immersed in it to the extent that there is no clear distinction between awareness and *the object of awareness*. The individual’s *being* is intertwined with the object of awareness. In an immersed state consciousness may be said to be “outside,” that is, with the sensations, with the experience, with that which is felt, known, and sensed. Alternatively, the opposite could be stated: everything happens within consciousness. Day-to-day experience happens within such a field of awareness, or meaning, where there is perhaps an identifiable distinction between the human being and its environment, but no perceived separation. When awareness is performed as part of a meditation practice, the practitioner enters this field of experience intentionally, blurring the lines between activity and receptivity (as indicated by “activity ≈ receptivity” in Figure 2).

When analyzed, however, awareness and object may seem to be fundamentally opposed. Awareness involves awareness *of* something, that is, an object (or a field of experience), while an object as such is never of something.^4^ Seemingly, awareness and object may never be fully unified, as the individual can point to at least some difference between the two. For example, an aspect of awareness must remain the same in experience, while specific objects always change (which is different from saying that awareness does not need an object). In self-awareness, however, awareness and object are fully unified. When an individual is aware of awareness, they are aware of their own production, their own activity of being aware (this is indicated in Figure 2 by “active = receptive”). Awareness of awareness includes the other activities in itself, as well as the object of awareness. Similarly, awareness always includes an object, awareness always includes some degree of observation, observation always includes some degree of release and focus, and so on. The other activities are, however, often performed subconsciously or indirectly. When focusing, for example, there is a subtle awareness of awareness performed, that is, a kind of monitoring of whether the intended activity of focus is performed or not. However, awareness of awareness does not require a concrete object or even field of experience as an object outside of itself. Although the grammatical structure of “awareness of …” implies that there is something that awareness is directed toward that is external to it (a subject separate from an object), when awareness is aware of itself, the subject/object structure dissolves into non-duality. The kind of awareness implied in “awareness of awareness” may be regarded as a kind of meta-awareness that is both sustained and non-propositional (Dunne et al., 2019), sometimes referred to as *samprayana*, a type of meta-awareness that is not focused on an object *per se*, but rather is an awareness of that intentional relation itself (Lutz et al., 2007, p. 504). We may to some extent distinguish between (i) awareness of awareness and (ii) awareness of the intentional relationship [having *x* (= not awareness itself) as an object of awareness]. However, (ii) depends on (i) if we conceive of awareness of awareness as a condition for maintaining awareness of the intentional relationship. Furthermore, when awareness is aware of itself, the distinction between (i) and (ii) collapses into non-duality.

Although, as noted previously, the number of objects in our model is unlimited, there may be limits as to how activities are combined with objects of meditation. For example, the practitioner may move the body, but the practitioner cannot (normally) move a visual impression. There may be exceptions to this, such as when the practice of the fire *kasiṇa* meditation results in perceived abilities of moving visual impressions (Ingram, 2018). Hence one will have to distinguish between what is logically impossible and what is typically impossible within non-meditative consciousness.

Figure 2 also contains two subcategories for each of focus, release, move, and imagine. Release, for example, can be divided into “let go” and “stay open.” These have not been added with the help of the 50 techniques considered here but are rather based primarily on further analysis and traditional literature. When releasing a mental object, we can distinguish between dropping content that is already there and remaining in a state of openness that is without any (coarse) content. In general, dropping is a momentary act, and staying open is a continuous process. However, *staying* open may be a matter of quickly dropping any subtle or coarse content that might appear. “Application,” one of the subcategories under “focus,” means to apply focus to an object. Alternatively, “sustaining” means to maintain continuous focus on an object. Like the two subcategories under “release,” “application” is momentary, while “sustaining” is continual. Here only “sustaining” may be understood to consist of, or include, “application.” In the same manner we can understand “create” and “sustain” (under “imagine”), and “place” and “maintain” (under “move”): for each of the subcategories, the ones on the left (“let go,” “apply,” etc.) are momentary, while the ones on the right (“stay open,” “sustain,” etc.) are continual. Additionally, the continual subcategories involve the momentary aspects as part of them, while the momentary subcategories are not necessarily continual. Hence these categories are not strictly opposites and are not united in the next, supraordinate, categories. This (and the fact that they were not derived from the 50 techniques) is indicated by the dotted lines. See Supplementary File 1 for an overview of which synonyms each of the meditative activities include and exclude.

Note that meditation instructions occasionally include descriptions of what the practitioner should avoid. Hence the meditative activities can be defined according to what the practitioner should do, and what the practitioner *should not do*. A classic example of this is Tilopa’s Six Words of Advice: “*Don’t recall. Let go of what has passed. Don’t imagine. Let go of what may come. Don’t think. Let go of what is happening now.*” et cetera. Here we see that a “do not” instruction may be formulated positively as well, that is, as “*let go*.” According to the different categories of meditative activities described above, instructions such as “don’t think” may be understood as a “release” instruction, or as do not “produce” “*thought*.” Furthermore, objects of meditation may be defined according to time (past, present, and future). “*Let go of what is happening now*” could then be understood as an instruction to release (or drop) any object currently present, and to “stay open” insofar as nothing is coarsely present. Furthermore, “object” included in Figure 1 may also be thought of as the general domain of experience. This then allows for the interpretation of instructions such as “maintain background awareness while focusing on object X.” That is, this would mean that the practitioner should be aware of the field of experience in which mental objects may appear, while focusing on a specific mental object. It is also possible to introduce transmodal fields of experience, such as time and space, though these might also be treated as objects of the awareness activities themselves. How objects of meditation are defined, or classified, is a complex matter and easily becomes entangled in questions of ontology and metaphysics. As we have stated earlier, is important to note that the “object” branch of the table can easily be expanded. If it is, this should be done based on phenomenological analysis aided by descriptions of meditation practices. See Supplementary File 2 for an attempt to break down typical descriptions [in this case provided by Upton and Brent (2019)] of meditation instructions into activities and objects.

Meditative activities may be modified in specific ways. For example, the practitioner may attend “closely,” observe “diligently,” or do “fast” noting (Ingram, 2018). We have not attempted here to create an overview over such modifiers. They may be understood either as a modification of the effort involved (e.g., diligently and fast) or they may be understood as one of the activities (e.g., attending closely may be understood to mean “focus” rather than “be aware of”). Furthermore, some meditation instructions may be understood as composites of multiple meditative activities. For example, consider the instruction “explore your experience” (Ray, 2018, p. 78). This may involve becoming aware of the field of experience, focusing on a sensation, observing tension, releasing tension, becoming aware of feelings, focusing on feelings, and so on. What the meditator performs when following such an instruction can be highly individualized, and without breaking it down to specific activities, it would not be possible to track the activities and their effects. The classification of activities suggested in this section will enable such research. Since meditation techniques often involve more than one activity, the overarching techniques will have to be defined. A suggestion of how use the classification system proposed here as a basis for defining techniques is provided in Supplementary File 2.

## Conclusion

We conclude with a definition of meditation: meditation is at least one of several intentional awareness activities such as observe, focus, release, produce, imagine, and move, underpinned and unified by the activity of awareness of awareness, performed in a formal or informal setting. The practice of these activities may result in altered states of consciousness, passing through stages of development, and ultimately endpoints of practices (e.g., “awakening,” “enlightenment”) (Reddy and Roy, 2019b). These states, stages, and experiences (or lack of experience) may be motivated by and interpreted within secular or spiritual frameworks.

This definition is, hopefully, not too broad, but also not too narrow. It is intended to encompass all aspects of mediation previously identified as well as aspects that have been overlooked. For example, “closed-eyes meditation” may be described as “maintain closed eyelids” (a form of *move* activity with the eyelids as object). Furthermore, this definition, and the classification system built into it, are intended to answer all the different challenges that arise when attempting to define mediation, such as whether it is a matter of doing or non-doing. Our definition draws on the strengths of different classification systems and is open to amendment. The way to test the validity and utility of this definition is to consider additional meditation instructions and descriptions of meditation practice. This system for defining meditation can be expanded by considering the experiential micro-dimensions of the meditative activities using applied phenomenological approaches [e.g., micro-phenomenology (Sparby, 2019b,c)]. Finally, our proposed definition can explain why both different and overlapping outcomes may arise from different meditative techniques, that is: different meditative techniques may involve some of the same activities, and will to a certain extent, this will always and necessarily be the case. Still, this definition accounts for varied outcomes that may be the result of different meditative techniques that emphasize different activities. Our activity-based definition promises to inform improved meditation training programs and interventions, and the study thereof, that will contribute to the reduction of suffering and the realization of the good.

## Data Availability Statement

The original contributions presented in the study are included in the article/Supplementary Material, further inquiries can be directed to the corresponding author.

## Author Contributions

TS wrote the initial draft. MS commented on and revised the initial draft. TS and MS revised the final draft. Both authors contributed to the article and approved the submitted version.

## Conflict of Interest

The authors declare that the research was conducted in the absence of any commercial or financial relationships that could be construed as a potential conflict of interest.

## Publisher’s Note

All claims expressed in this article are solely those of the authors and do not necessarily represent those of their affiliated organizations, or those of the publisher, the editors and the reviewers. Any product that may be evaluated in this article, or claim that may be made by its manufacturer, is not guaranteed or endorsed by the publisher.

## Footnotes

1 However, there may be a stronger connection between the mental activities of meditation and their spiritual (awakening) effects than walking and the potential spiritual effects of a pilgrimage.

2 There is an open question as to what extent *flow* and mindfulness or meta-awareness, in the sense of being conscious of what is going on in the present moment, exclude one another. One prior study has maintained that this is indeed the case, that is, that these processes exclude one another (Sheldon et al., 2015). However, while mindfulness/meta-awareness may potentially disturb certain flow experiences, maintaining continual introspective awareness is both recommended for entering deeper states of meditation such as jhāna and has been reported as part of the experience of such flow states (Yates et al., 2015; Sparby, 2019c). Thus, future research should endeavor to further disentangle interactions among flow and meditation.

3 Note that perhaps apart from *move*, the activities may be regarded as mental activities. “Move” is physical when the physical body is moved, but not when one tries to move “energies” or “sensations” around in the body. Arguably, all activities of an agent are mental in origin. When moving the physical body, the correct analysis is that it is a mental activity directed at the physical body. Sidestepping this issue, one may refer to the activities involved as “awareness activities.”

4 An object may be a sign or a picture, and hence, in a sense be about *something*. However, an object becomes a sign, or a picture, through a *subject*.

## References

[B1] American Psychiatric Association (2013). *Diagnostic and Statistical Mental Disorders. DSM-5. American Psychiatric Association.* Washington, DC: American Psychiatric Publishing. 10.1176/appi.books.9780890425596

[B2] Anālayo B. (2018). *Satipatthana Meditation. A Practice Guide.* Cambridge: Windhorse Publications.

[B3] Anālayo B. (2020). Buddhist Antecedents to the Body Scan Meditation. *Mindfulness* 2020:8. 10.1007/s12671-019-01259-8

[B4] Aristotle (1984). “The Complete Works of Aristotle,” in *The Revised Oxford Translation II*, ed. Barnes J. (Princeton, NJ: Princeton University Press),

[B5] Baker S. H. (2021). A Monistic Conclusion to Aristotle’s Ergon Argument: The Human Good as the Best Achievement of a Human. *Archiv Fur Geschichte Der Philosop.* 103 373–403. 10.1515/agph-2018-0031

[B6] Bodhi B. (2000). *The noble eightfold path. Way to the End of Suffering.* Seattle: BPS Pariyatti Editions.

[B7] Bortoft H. (2013). *The Wholeness of Nature. Goethe’s Way Toward a Science of Conscious Participation in Nature.* Edinburgh: Floris Books.

[B8] Brahm A. (2014). *Mindfulness, Bliss, and Beyond. A Meditator’s Handbook.* Somerville: Wisdom Publications.

[B9] Britton W. B. Davis J. H. Loucks E. B. Peterson B. Cullen B. H. Reuter L. (2018). Dismantling Mindfulness-Based Cognitive Therapy: Creation and validation of 8-week focused attention and open monitoring interventions within a 3-armed randomized controlled trial. *Behav. Res. Therapy* 101 92–10. 10.1016/j.brat.2017.09.010 29106898 PMC5801080

[B10] Brown D. (2006). *Pointing Out the Great Way. The Stages of Meditation in the Mahamudra Tradition.* Boston, MA: Wisdom Publications.

[B11] Bryant E. F. (2009). *The Yoga Sūtras of Patañjali.* New York, NY: North Point Press.

[B12] Buddhaghosa B. (2010). *Visuddhimagga.* Kandy: Buddhist Publication Society.

[B13] Catherine S. (2008). *Focused and Fearless.* Boston, MA: Wisdom Publications.

[B14] Catherine S. (2011). *Wisdom Wide and Deep. A Practical Handbook for Mastering Jhāna and Vipassanā.* Somerville, MA: Wisdom Publications.

[B15] Chlup R. (2012). *Proclus. An Introduction.* Cambridge, MA: Cambridge University Press. 10.1017/CBO9781139028042

[B16] Dahlberg K. (2006). The essence of essences - The search for meaning structures in phenomenological analysis of lifeworld phenomena. *Int. J. Qualit. Stud. Health Well Being* 2006:17482620500478405. 10.1080/17482620500478405

[B17] Dasanayaka N. N. Sirisena N. D. Samaranayake N. (2021). The effects of meditation on length of telomeres in healthy individuals: a systematic review. *Systemat. Rev.* 10:151. 10.1186/s13643-021-01699-1 34020720 PMC8139075

[B18] Didonna F. (2020). *Mindfulness-Based Cognitive Therapy for OCD: A Treatment Manual. Mindfulness-based cognitive therapy for OCD: A treatment manual.* New York, NY: The Guilford Press.

[B19] Dunne J. D. Thompson E. Schooler J. (2019). Mindful meta-awareness: sustained and non-propositional. *Curr. Opin. Psychol.* 28 307–311. 10.1016/j.copsyc.2019.07.003 31374535

[B20] Eddy G. (2019). Deity practice in the FPMT: understanding the nature of the Tibetan Buddhist deity from the Western practitioner’s perspective. *Cult. Relig.* 1 169–191. 10.1080/14755610.2019.1627376

[B21] Farias M. Maraldi E. Wallenkampf K. C. Lucchetti G. (2020). Adverse events in meditation practices and meditation-based therapies: a systematic review. *Acta Psychiatr. Scand.* 142 374–393. 10.1111/acps.13225 32820538

[B22] Goyal M. Singh S. Sibinga E. M. S. Gould N. F. Rowland-Seymour A. Sharma R. (2014). Meditation programs for psychological stress and well-being. *JAMA Internal Med.* 174:357. 10.1001/jamainternmed.2013.13018 24395196 PMC4142584

[B23] Grayson M. L. Cosgrove S. E. Crowe S. M. Hope W. McCarthy J. S. Mills J. (2017). *Kucers’ the use of antibiotics: A clinical review of antibacterial, antifungal, antiparasitic, and antiviral drugs, seventh edition. Kucers the Use of Antibiotics: A Clinical Review of Antibacterial, Antifungal, Antiparasitic, and Antiviral Drugs*, 7th Edn. Baltimore, MD: Johns Hopkins University, 1–4841.

[B24] Haider T. Dai C. L. Sharma M. (2021). Efficacy of Meditation-Based Interventions on Post-Traumatic Stress Disorder (PTSD) Among Veterans: A Narrative Review. *Adv. Mind Body Med.* 35 16–24. 33513582

[B25] Hegel G. W. F. (1834). *Wissenschaft der Logik. Georg Wilhelm Friedrich Hegel’s Werke / Vollständige Ausgabe durch einen Verein von Freunden des Verewigten.* Berlin: Duncker und Humblot.

[B26] Hirshberg M. J. Goldberg S. B. Rosenkranz M. Davidson R. J. (2020). Prevalence of harm in mindfulness-based stress reduction. *Psychol. Med.* [Preprint]. 10.1017/S0033291720002834 32807249 PMC7889774

[B27] Holton R. (2009). *Willing, Wanting, Waiting.* Oxford: Clarendon Press, 10.1093/acprof:oso/9780199214570.001.0001

[B28] Hoyningen-Huene P. (2016). *Systematicity. The Nature of Sience.* Oxford: Oxford University Press.

[B29] Husserl E. (1976). *Ideen zu einer reinen Phänomenologie und Phänomenologischen Philosophie.* Den Haag: Nijhoff.

[B30] Ingram D. (2018). *Mastering the Core Teachings of the Buddha: An Unusually Hardcore Dharma Book.* London: AEON.

[B31] Kabat-Zinn J. (2018). *Meditation Is Not What You Think It Is.* London: Piatkus.

[B32] Komarovski Y. (2015). *Tibetan Buddhism and Mystical Experience.* Oxford: Oxford University Press. 10.1093/acprof:oso/9780190244958.001.0001

[B33] Kreplin U. Farias M. Brazil I. A. (2018). The limited prosocial effects of meditation. A systematic review and meta-analysis. *Sci. Rep.* 8:2403. 10.1038/s41598-018-20299-z 29402955 PMC5799363

[B34] Kunga Tenzin N. (2014). *The Royal Seal of Mahamudra. Volume One: A Guidebook for the Realization fo Coemergence.* Boston, MA: Snow Lion.

[B35] Latham N. (2015). Meditation and Self-Control. *Philosop. Stud.* 10 1–22.

[B36] Lindahl J. (2017). Somatic Energies and Emotional Traumas: A Qualitative Study of Practice-Related Challenges Reported by Vajrayāna Buddhists. *Religions* 8:153. 10.3390/rel8080153

[B37] Lindahl J. R. Britton W. B. (2019). I have this feeling of not really being here: Buddhist meditation and changes in sense of self. *J. Conscious. Stud.* 26 157–183.

[B38] Lindahl J. R. Fisher N. E. Cooper D. J. Rosen R. K. Britton W. B. (2017). The varieties of contemplative experience: A mixed-methods study of meditation-related challenges in Western Buddhists. *PLoS One* 12:e0176239. 10.1371/journal.pone.0176239 28542181 PMC5443484

[B39] Lott D. T. Yeshi T. Norchung N. Dolma S. Tsering N. Jinpa N. (2021). No Detectable Electroencephalographic Activity After Clinical Declaration of Death Among Tibetan Buddhist Meditators in Apparent Tukdam, a Putative Postmortem Meditation State. *Front. Psychol.* 11:599190. 10.3389/fpsyg.2020.599190 33584435 PMC7876463

[B40] Lundh L. G. (2020). Experimental Phenomenology in Mindfulness Research. *Mindfulness* 11 493–506. 10.1007/s12671-019-01274-9

[B41] Lutz A. Dunne J. D. Davidson R. J. (2007). Meditation and the Neuroscience of Consciousness. *Cambridge Handb. Conscious.* 2007 499–551. 10.1017/CBO9780511816789.020

[B42] Lutz A. Jha A. P. Dunne J. D. Saron C. D. (2015). Investigating the Phenomenological Matrix of Mindfulness-Related Practices From a Neurocognitive Perspective. *Am. Psychol.* 70 632–658. 10.1037/a0039585 26436313 PMC4608430

[B43] Matko K. Sedlmeier P. (2019). What Is Meditation? Proposing an Empirically Derived Classification System. *Front. Psychol.* 10:2276. 10.3389/fpsyg.2019.02276 31681085 PMC6803504

[B44] Matko K. Ott U. Sedlmeier P. (2021). What Do Meditators Do When They Meditate? Proposing a Novel Basis for Future Meditation Research. *Mindfulness* 12 1791–1811. 10.1007/s12671-021-01641-5

[B45] Moustakas C. E. (2010). *Phenomenological Research Methods.* Thousand Oaks, CA: Sage Publications.

[B46] Noë A. (2012). *Varieties of Presence.* Cambridge, MA: Harvard University Press. 10.4159/harvard.9780674063013

[B47] Ospina M. B. Bond K. Karkhaneh M. Tjosvold L. Vandermeer B. Liang Y. (2007). *Meditation Practices for Health: State of the Research. Evidence Report/Technology Assessment*, Vol. 155. Rockville, MD: Agency for Health Care Research and Quality, 1–263. PMC4780968 17764203

[B48] Padoux A. (1992). *Vāc: The Concept of the Word in Selected Hindu Tantras.* Albany: New York Press.

[B49] Perlman D. M. Salomons T. V. Davidson R. J. Lutz A. (2010). Differential Effects on Pain Intensity and Unpleasantness of Two Meditation Practices. *Emotion* 10 65–71. 10.1037/a0018440 20141303 PMC2859822

[B50] Petitmengin C. Van Beek M. Bitbol M. Nissou J.-M. Roepstorff A. (2017). What is it Like to Meditate? Methods and Issues for a Micro-phenomenological Description of Meditative Experience. *J. Conscious Stud.* 24 170–198.

[B51] Przyrembel M. Singer T. (2018). Experiencing meditation – Evidence for differential effects of three contemplative mental practices in micro-phenomenological interviews. *Conscious. Cognit.* 62 82–101. 10.1016/j.concog.2018.04.004 29747119

[B52] Ray R. (2018). *The Practice of Pure Awareness: Somatic Meditation for Awakening the Sacred.* Boulder: Shambhala.

[B53] Reddy J. S. K. Roy S. (2018). Commentary: Patanjali and neuroscientific research on meditation. *Front. Psychol.* 6:915. 10.3389/fpsyg.2018.00248 29535670 PMC5835223

[B54] Reddy J. S. K. Roy S. (2019a). The role of one’s motive in meditation practices and prosociality. *Front. Hum. Neurosci.* 13:48. 10.3389/fnhum.2019.00048 30814944 PMC6381069

[B55] Reddy J. S. K. Roy S. (2019b). Understanding meditation based on the subjective experience and traditional goal: Implications for current meditation research. *Front. Psychol.* 10:1827. 10.3389/fpsyg.2019.01827 31496967 PMC6712509

[B56] Reddy J. S. K. Roy S. (2020). Meditation-Induced Prosociality: An Integral Analysis Based on Traditional and Scientific Understanding. *J. Psychosoc. Res.* [Preprint]. 10.32381/JPR.2020.15.02.10

[B57] Rinpoche A. Graboski A. (2012). *Journey to Certainty. The Quintessence of the Dzogchen View.* Boston, MA: Wisdom Publications.

[B58] Rose K. (2016). *Yoga, Meditation, and Mysticism.* London: Bloomsbury Academic.

[B59] Saini G. K. Haseeb S. B. Taghi-Zada Z. Ng J. Y. (2021). The effects of meditation on individuals facing loneliness: a scoping review. *BMC Psychol.* 9:88. 10.1186/s40359-021-00585-8 34022961 PMC8140565

[B60] Sayadaw M. (2016). *Manual of Insight.* Somerville, MA: Wisdom Publications.

[B61] Schlosser M. Sparby T. Vörös S. Jones R. Marchant N. L. (2019). Unpleasant meditation-related experiences in regular meditators: Prevalence, predictors, and conceptual considerations. *PLoS One* 14:e0216643. 10.1371/journal.pone.0216643 31071152 PMC6508707

[B62] Schmidt S. (2014a). “Opening Up Meditation for Science: The Development of a Meditation Classification System,” in *Meditation - Neuroscientific Approaches*, eds Schmidt S. Walach H. (Cham: Springer). 10.1007/978-3-319-01634-4_8

[B63] Schmidt S. (2014b). “The development of a meditation classification system,” in *Meditation – neuroscientific approaches and philosophical implications*, Vol. 2 eds Schmidt S. Walach H. (Cham: Springer).

[B64] Sears S. Kraus S. (2009). I think therefore I om: Cognitive distortions and coping style as mediators for the effects of mindfulness meditation on anxiety, positive and negative affect, and hope. *J. Clin. Psychol.* 65 561–573. 10.1002/jclp.20543 19241400

[B65] Sedlmeier P. Theumer J. (2020). Why Do People Begin to Meditate and Why Do They Continue? *Mindfulness* 11 ages1527–ages1545. 10.1007/s12671-020-01367-w

[B66] Sheldon K. M. Prentice M. Halusic M. (2015). The Experiential Incompatibility of Mindfulness and Flow Absorption. *Soc. Psychol. Pers. Sci.* 6 276–283. 10.1177/1948550614555028

[B67] Smith B. (2015). *The Dingo Debate: Origins, Behaviour and Conservation.* Melbourne: Csiro Publishing. 10.1071/9781486300303

[B68] Snyder S. Rasmussen T. (2009). *Practicing the Jhanas. Traditional concentration meditation as presented by the Venerable Pa Auk Sayadaw.* Boston, MA: Shambhala.

[B69] Sparby T. (2014). *Hegel’s Conception of the Determinate Negation.* Leiden: Brill, 10.1163/9789004284616

[B70] Sparby T. (2015). Investigating the depths of consciousness through meditation. *Mind Matter* 13 213–240.

[B71] Sparby T. (2019a). Body, Soul, and Spirit. A Qualitative Study of Anthroposophic Meditation. *Religions* 11:314. 10.3390/rel11060314

[B72] Sparby T. (2019b). Fear, Bliss, and Breathing Changes during Meditation. A Case Study of a Transformative Experience. *Mind Matter* 17 7–35.

[B73] Sparby T. (2019c). Phenomenology and Contemplative Universals: The Meditative Experience of Dhyāna, Coalescence or Access Concentration. *J. Conscious. Stud.* 26 130–156.

[B74] Sparby T. Ott U. (2018). A qualitative study of motivations for meditation in anthroposophic practitioners. *PLoS One* 13:e0203184. 10.1371/journal.pone.0203184 30212522 PMC6136727

[B75] Strawson G. (2003). Mental ballistics or the involuntariness of spontaneity. *Proc. Aristotelean Soc.* 103 227–256. 10.1111/j.0066-7372.2003.00071.x

[B76] Sumantry D. Stewart K. E. (2021). Meditation, Mindfulness, and Attention: a Meta-analysis. *Mindfulness* 12 1332–1349. 10.1007/s12671-021-01593-w

[B77] Upton C. L. Brent M. (2019). Meditation and the scope of mental action. *Philosop. Psychol.* 32 52–71. 10.1080/09515089.2018.1514491

[B78] Valk S. L. Bernhardt B. C. Trautwein F. M. Böckler A. Kanske P. Guizard N. (2017). Structural plasticity of the social brain: Differential change after socio-affective and cognitive mental training. *Sci. Adv.* 3:e1700489. 10.1126/sciadv.1700489 28983507 PMC5627980

[B79] Vos J. (2016). “Working with Meaning in Life in Mental Health Care: A Systematic Literature Review of the Practices and Effectiveness of Meaning-Centred Therapies,” in *Clinical Perspectives on Meaning*, eds Russo-Netzer P. Schulenberg S. E. Batthyany A. (Berlin: Springer), 59–87. 10.1007/978-3-319-41397-6_4

[B80] Wallace A. B. Houshmand Z. (2010). *The Four Immeasurables: Practices to Open the Heart.* Ithaca, NY: Snow Lion.

[B81] Wallace B. A. (2006). *The attention revolution: Unlocking the power of the focused mind.* Boston, MA: Wisdom Publications.

[B82] Walsh R. Shapiro S. L. (2006). The meeting of meditative disciplines and western psychology: A mutually enriching dialogue. *Am. Psychol.* 61 227–239. 10.1037/0003-066X.61.3.227 16594839

[B83] Wittgenstein L. (1953). *Philosophical Investigations.* Oxford: Blackwell, 10.1017/S0031819100034616

[B84] Worth N. (2021). “One’s Own Body of Pure Channels and Elements”: The Teaching and Practice of Tibetan Yoga at Namdroling. *Religions* 12:404. 10.3390/rel12060404

[B85] Yates J. Immergut M. Graves J. (2015). *The Mind Illuminated. A Complete Meditation Guide Integrating Buddhist Wisdom and Brain Science.* New York, NY: Atria Books.

[B86] Zhang Q. Wang Z. Wang X. Liu L. Zhang J. Zhou R. (2019). The effects of different stages of mindfulness meditation training on emotion regulation. *Front. Hum. Neurosci.* 13:208. 10.3389/fnhum.2019.00208 31316361 PMC6610260

[B87] Zhang Z. Luh W. M. Duan W. Zhou G. D. Weinschenk G. Anderson A. K. (2021). Longitudinal effects of meditation on brain resting-state functional connectivity. *Sci. Rep.* 11:11361. 10.1038/s41598-021-90729-y 34059702 PMC8166909

