# Supplementary material for: Defining Meditation: Foundations for an Activity-Based Phenomenological Classification System
Source: Front Psychol. 2022 Jan 28;12:795077. doi: 10.3389/fpsyg.2021.795077 (PMC8832115; doi:10.3389/fpsyg.2021.795077)
Supplement: Supplementary file 1 [file Presentation_1.pdf]

Supplement 1: Inclusions, Exclusions and Comments on the Different Activities

Activity	Inclusions, Exclusions and Comments
**Awareness of Awareness**	Includes: Meta-cognition.<br><br>Comment: Awareness of awareness may also be differentiated. For example, one may be aware of being aware of pain. This is a case of awareness of awareness taking the activity of awareness of pain as an object.
**Awareness**	Includes: Sense. Feel. Know. Recognize. Apprehend. Connect with. Listen.<br><br>Excludes: Awareness of distinct objects external to the subject, such as objects of the physical senses, are "observe".<br><br>Comment: "Awareness" may be taken to refer to activities where the emphasis is on unity/non-duality in relation to the object.
**Observe**	Includes: Watch. See. Listen. Note. Notice. Perceive. Discern. Detect. Behold. Distinguish. Detect.
**Focus**<br>Subcategories: Apply. Sustain.	Includes: Attend. Concentrate. Zoom in on. Centre. Anchor. Merge.
**Release**<br>Subcategories: Let go. Stay open.	Includes: Relax. Loosen. Expand. Decrease. Allow. Free. Detach. Open. Accept.
**Produce**	Includes: Energize. Intensify. Increase. Make. Combine. Generate.
**Imagine**<br>*Subcategories:* Create. Prolong.	Includes: Picture. Visualize.
**Move**<br>*Subcategories*: Place. Maintain.	Hold. Walk. Shift. Change.

General Notes:

Activities can take other activities as their object.

Each activity can be *modified* by adverbs (strongly/gently, fast/slow, etc.).

Meditation instructions may be formulated either as a "do this" or a "do not do this" instruction.

The objects of the activities may be either specific (e.g. the breath), modalities of experience (e.g. the visual field) or the field of experience in general (which may sometimes be qualities, e.g. as "inner" and outer"). For example, "listen inside" may be taken to mean "be aware of the *field of inner experience*".

Terms designated as "inclusions" are examples and not comprehensive lists. Included terms may potentially be analyzed in a manner where they represent a combination of activities.
