# Supplementary material for: Defining Meditation: Foundations for an Activity-Based Phenomenological Classification System
Source: Front Psychol. 2022 Jan 28;12:795077. doi: 10.3389/fpsyg.2021.795077 (PMC8832115; doi:10.3389/fpsyg.2021.795077)
Supplement: Supplementary file 2 [file Presentation_2.pdf]

Supplement 2: Defining Techniques Based on Activities

This supplement suggests how one can approach the relationship between single activities and elaborate techniques. Breaking down techniques into activates is vital when investigating whether techniques that are named differently are similar or the same at the level of activity. Furthermore, defining techniques based on what activities are performed is vital when investigating meditation effects experimentally, as this enables linking the effects to what is actually performed during the meditation.

A problem that arises when defining meditation according to activities is that some meditative activities may be performed occasionally (such as maintaining background awareness) while others may be performed all the time (maintaining a body posture). What if a practitioner performs a focused attention meditation, but in practice their mind mostly wandered? This could lead to a false association of effects, that is, an unintended activity (e.g. mind-wandering) may be associated with certain effects while the investigators believe that a focus meditation was performed. Furthermore, it would be problematic for a practitioner to track precisely the kind of activities that they perform, especially over long periods, since continually reporting on whether one is performing an activity is likely to disturb the process of meditation.

These problems can be resolved in the following way. Techniques can be defined as collections of activities, some of which are primary, others of which are secondary. We define primary activities as those that one intends to perform as consistently as possible. In order to provide further precision, each activity can then be rated according to whether the practitioner never, rarely, sometimes, often, most of the time, or always performed that activity (or any other way of scale definition). If the practitioner never performed any of the primary activities, it cannot be said that they performed the intended meditation. What is considered the primary dimensions of the meditation technique may be negotiated by the individuals involved (researchers, meditators, teachers, etc.), the traditional and contemporary meditation handbooks, and the particular context of practice. The primary activities may be linked to what these individuals justifiably believe to be the most important aspects for inducing intended effects. Secondary activities may be understood as activities that support the main activities, or support the meditator practice when encountering difficulties with practice.

Another pragmatic approach to classifying meditative practice is to define a meditation technique as that activity which one intends to perform more than half of the time, or, relatedly, the activity that is emphasized. What is emphasized may be defined, for example, as that which the practitioner mostly intends to do (e.g., focus), in contrast to other activities that the

practitioner intends to do rarely, or to avoid entirely (e.g., reflecting on the reason[s] for a specific thought appearing repeatedly). This notion is of particular importance in a retreat settings, where retreatants are investigated over many days and may potentially make use of other, complementary techniques that they have generated habituated reliance from prior meditation experience. Furthermore, defining meditation techniques according to emphasis is in accordance with the general idea of the present theory of meditation, namely: that meditative activities necessarily involve other activities, although these other activities may not be emphasized and/or performed more or less subconsciously or indirectly.

Meditative techniques can be formulated as templates that include the primary and secondary activities, which may include "if"-conditions (e.g.: if attention wanders, return to the object of focus). These templates may be written in plain language including indicated activities and meditation objects clearly identified for each instruction.

Example Definitions of Techniques

The descriptions of each meditation technique is based on (Upton & Brent, 2019). The instructions are written in plain text. The analysis is given above each instruction in italics. Red color indicates activity and blue color indicates object.

Focus Awareness (FA)

[PRIMARY EMPHASIS]:
Apply focus on object x
Introduces attentional focus

Sustain focus on object x
Maintain attentional focus

[SECONDARY EMPHASIS]:
Be aware of the field of experience (all possible objects)
Maintains awareness of her background

Be aware of not focusing on object x
Notice when/if her focus has shifted from positive anchor

If aware of not focusing on object x, apply focus on object x
Shifts focus back to the chosen positive anchor

Release thoughts
Removes automatically generated cognitive content

Open Monitoring (OM)

[PRIMARY EMPHASIS]:
Release focus on any object
Remove focus

Observe the field of experience (all possible objects)
[Lacking in original instruction]

[SECONDARY EMPHASIS]:
Be aware of not observing the field of experience
Notices when monitoring becomes distracted

If aware of not observing the field of experience, observe the field of experience
Shift back to a state of awareness with no focus

Release thoughts
Remove automatically generated cognitive content

Loving-Kindness Meditation (LKM)

[PRIMARY EMPHASIS]:

Produce thoughts of loving-kindness about being x
Introduces cognitive content

Produce feelings of loving-kindness towards being x
Introduce an affective state

[SECONDARY EMPHASIS]:

Release hateful emotions towards being x
Remove affective state

Release all reactions related to hateful feelings towards being x
De-intensify an affective state

Release all other content
Removes content

Focus on thoughts and feelings of loving-kindness about being x
Intensify an affective state or
